# Specialization and integration of brain responses to object recognition and location detection

**DOI:** 10.1002/brb3.27

**Published:** 2012-01

**Authors:** Mark R Pennick, Rajesh K Kana

**Affiliations:** Department of Psychology, University of Alabama at BirminghamAlabama

**Keywords:** Dorsal, fMRI, functional connectivity, integration, specialization, ventral

## Abstract

Visual information is processed in the brain primarily through two distinct pathways, the dorsal and the ventral visual streams. The present functional magnetic resonance imaging study investigated the specialization and integration of dorsal and ventral streams using tasks of object recognition and location detection. The study included 22 healthy adult volunteers who viewed stimuli consisting of grayscale photographs of common household objects presented in blocked design. Participants were asked to either recognize an object or to locate its position. While the location detection task elicited greater activation in the dorsal visual stream, recognizing objects showed greater activation in the middle occipital gyri, left inferior temporal gyrus, and in the left inferior frontal gyrus. The integration between dorsal and ventral brain areas was stronger during location detection than during object recognition. In addition, a principal components analysis found preliminary evidence for a group of regions, such as frontal and parietal cortex, working together in this task. Overall, the results of this study indicate the existence of specialized modules for object recognition and location detection, and possible interactions between areas beyond the visual cortex that may play a role in such tasks.

## Introduction

Few topics in neuroscience seem to have attracted more attention than vision, perhaps due to its complexity, its importance to humans, the relatively vast cortical space devoted to it, and to the extensive and illuminating research done in monkey visual cortex. Although the occipital cortex along with large portions of the temporal and parietal lobes make up the visual information processing system (Goodale and Milner 1992), there is evidence for areas as distant as the orbitofrontal cortex (OFC) playing a key role in tasks, such as object recognition, with brain activity seen as early as 80 msec after stimulus presentation (Bullier 2001; Bar et al. 2006). This illustrates the role of diffuse networks in visual information processing, possibly rekindling the debate in neuroscience on cortical specialization and integration.

One of the earliest models of visual processing, which continues to demonstrate distinct merit, is the dorsal and ventral visual stream model (Ungerleider and Mishkin 1982). Developed on the basis of extensive research on monkeys, this model showed that while lesions in the parietal lobe of the brain lead to deficits in location detection (the *where* pathway), lesions in the inferior temporal areas result in deficits in object recognition (the *what* pathway). Thus, the model suggested distinct modules that may underlie specialized tasks and hypothesized a segregation of magnocellular and parvocellular inputs to the dorsal and ventral visual streams, respectively. This line of research paved the foundation on which a wide body of research has built upon and updated over the years, of late with neuroimaging techniques, such as positron emission topography and functional magnetic resonance imaging (fMRI). A recent fMRI study examined the dorsal and ventral stream response to varying identities and locations of objects (Valyear et al. 2006), finding increased activity in the ventral stream in response to changing identities of objects (and no difference in the dorsal stream), and greater activity in the dorsal stream in response to change in object locations. There are also several other studies that support this functional independence (Cavina–Pratesi et al. 2007; Bruno et al. 2008; Shmuelof and Zohary 2008), reminiscent of the findings from (Ungerleider and Mishkin 1982).

Despite the evidence for functional independence, there are also findings that support visual information processing being relatively more integrative. For example, object perception may elicit significant activation in the lateral occipital complex and the posterior parietal cortex suggesting that the perception of an object may involve reliance on higher order visual areas in both dorsal and ventral streams (Konen and Kastner 2008). In addition, several fMRI studies provide evidence for the communication between the dorsal and ventral streams during tasks that were theorized to activate only one visual stream (Schenk and Milner 2006; Mahon et al. 2007; Ploran et al. 2007). This pattern was also found in studies of color discrimination, arguably one of the most segregated visual tasks (Claeys et al. 2004). Another study used effective connectivity, the causal influence of one region on another (Friston 1994), to examine the interaction of parietal and temporal lobes during a task of spatial and object processing (Buchel et al. 1999). In their task, the effective connectivity increased as a function of learning, showing that the interaction of these brain areas during object recognition and location detection was instrumental in accomplishing the task. Overall, evidence from these studies points to a possible dialogue between different functionally specialized modules during visual perception.

Yet, another line of evidence for integration of different streams pertains to the idea that higher order processing areas, such as the motion sensitive visual cortex, receive feed-forward visual information and send feedback signals fast enough for primary visual cortex to integrate that information into a cohesive representation (Bullier 2001). In this way, areas V1 and V2 act as “blackboards” where information from higher order areas, even as distant as the OFC, is collected and integrated. This is demonstrated by event-related potentials at 50 msec faster in the OFC than in the temporal lobes after the presentation of a visual stimulus (Bar et al. 2006). Thus, the interaction of frontal and visual areas seems instrumental in accomplishing visual tasks, and possibly more so in visual tasks with a cognitive component. From this perspective, the visual system seems to operate globally at first, before beginning to make more local interpretations.

The different lines of evidence for the segregated and integrated models of visual information processing pose an interesting problem that has relevance to the delicate balance of specialization and integration in brain organization and development. The primary objective of the present fMRI study is to investigate the extent to which modular and network approaches can explain visual information processing in the context of tasks of object recognition and location detection. Neither, if examined in isolation, may provide a complete answer. Our approach focuses on examining activation as well as the functional synchronization of activated brain areas while accomplishing these tasks. We predict specialized areas, such as the dorsal and ventral visual streams, working in concert with each other and with other spatially distant brain areas, such as the frontal lobe, to solve tasks of object recognition and location detection.

## Materials and Methods

### Participants

The study consisted of 22 healthy participants (right-handed; mean age, 20.9 years; 15 males and seven females) recruited through the *Introduction to Psychology* course (PY101) of the Department of Psychology at the University of Alabama at Birmingham (UAB). The verbal, performance, and full-scale intelligence quotients (VIQ, PIQ, and FSIQ, respectively) of the participants were measured using The Kaufman Brief Intelligence Test (KBIT-2) (Kaufman and Kaufman 2004). Participants were excluded from the study if they were left-handed, reported any neurological disorders, reported claustrophobia, a body mass index exceeding 34, had metal implants or history of working with metal, kidney disease, diabetes, hypertension, anemia, sickle cell disease, or if they were taking psychotropic medications. All participants completed an informed consent that was approved by the UAB Institutional Review Board.

Although fMRI scans were acquired for 30 participants, the final analysis of 22 participants only included the scans that were not corrupted by artifacts and/or scored more than two standard deviations away from the mean accuracy for the group. These accuracy scores were determined by correct answers of locations or object identities based on button presses that indicated responses to text that accompanied the pictures. The cut off for head motion was set as 2 mm. Each participant's data were examined for continuous motion, intermittent spikes, and drifts in *x*, *y*, and *z* directions after the realignment step during data preprocessing. In addition, the Artifact Detection Tools (ART) software was used to identify global mean signal intensity and motion outliers (Gabrieli Lab, 2009; Whitfield-Gabrieli, Mozes, & Castanon, MIT). Yet, another step was to measure the temporal signal-to-noise ratio of each participant's data and a minimum cut off was kept at 40. Thus, the decision to not include a subject's data in the group analysis was made by taking into consideration all these aspects.

### Materials

**Figure 1:**
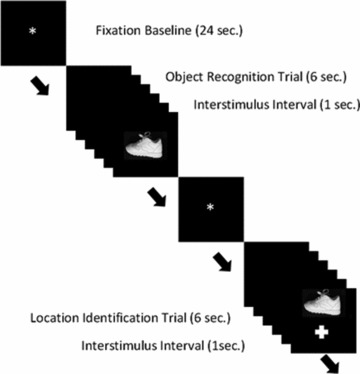
A pictorial representation of the timing of the trials and the types of stimuli and conditions presented in the study.

The stimuli were created using grey-scale photographs of a series of small common household objects against a black background. The objects generally fit into the categories of miniature animals, children's toys, kitchen objects, and clothing items. Each stimulus presented in the experiment was unique and the presentation of the blocks was pseudorandomized with two tasks (four blocks of object recognition and four blocks of location detection) and a fixation baseline. In the object task, participants recognized a given object and chose the appropriate name for it from a list of four alternatives, and in the location detection task, they detected the location of a given object relative to a cross at the center of the screen. The objects were positioned in four possible locations (left, right, above, and below) relative to the cross. Participant responses were recorded using fiber optic buttons. The recorded responses provided the reaction time and performance accuracy data for the object and location tasks. For both tasks, each item was presented for 6 sec during which the participant chose the correct answer. Each block consisted of six pictures with an interstimulus interval of 1 sec (see Fig. 1).

### Data acquisition and analysis

All participants practiced the experiment on a laptop computer before the scanning session started. While in the scanner, the software E-Prime 1.2 (Psychology Software Tools, Pittsburgh, PA) was used to present the stimuli. An IFIS (Integrated Functional Imaging System, Invivo Corporation, Orlando, FL) interface projected the data onto a screen behind the participant's head that was viewed using a mirror. Images were acquired using a 3T Siemens Allegra head-only scanner (*Siemens* Medical Inc., Erlangen, Germany) housed at the Civitan International Research Center, UAB. Structural images were acquired using high-resolution T1-weighted scans using a 160 slice 3D MPRAGE volume scan with a TR = 200 msec, TE = 3.34 msec, flip angle = 7, Field of View = 25.6 cm, 256 × 256 matrix size, and 1-mm slice thickness. To record functional imaging data, a single-shot gradient-recalled echo-planar pulse sequence was used which offers the advantage of rapid image acquisition (TR = 1000 msec, TE = 30 msec, flip angle = 60 degrees, FoV (Field Of View) = 24 cm, matrix 64 × 64). This sequence covers most of the cortex (17 5-mm thick slices with a 1 mm gap were acquired in an oblique-axial orientation) in a single cycle of scanning (one TR) with an in-plane resolution of 3.75 × 3.75 × 5 mm.

The data were preprocessed and statistically analyzed using SPM2 (Wellcome Department of Cognitive Neurology, London, U.K.). Images were corrected for slice acquisition timing, motion-corrected, and normalized to the MNI (Montreal Neurological Institute) template, re-sampled to 2-mm^3^ voxels, and smoothed with an 8-mm FWHM (Full Width Half Maximum) filter. Statistical analyses were performed on individual data by using the general linear model, while group analysis used random-effects models. Areas of statistically significant activation were determined using a *t*-statistic on a voxel-by-voxel basis. For statistical significance, the data were examined using family-wise error corrected for multiple comparisons (*P* < 0.05) for the contrasts between the tasks with fixation. For direct contrasts between conditions, we applied Monte Carlo simulations to the data using AlphaSim in AFNI (Analysis of Functional NeuroImages) to determine the minimum number of voxels in each cluster to be equivalent to the level of statistical significance at a family-wise error corrected threshold of *P* < 0.05. Based on this simulation, an uncorrected threshold of *P*= 0.001 and an extent threshold of 88 2-mm^3^ voxels was used.

### Functional connectivity analysis

Functional connectivity (the synchronization of brain activation between regions) was computed (separately for each participant) as a correlation between the average time course of all the activated voxels in each member of a pair of ROIs. Sixteen functional ROIs (Region of Interest) (supplementary motor area, SMA; left inferior parietal lobule, LIPL; right inferior parietal lobule, RIPL; left middle frontal gyrus, LMFG; left precentral, LPRCN; medial prefrontal cortex, MPFC; right thalamus, RTHAL; left thalamus, LTHAL; left inferior temporal gyrus, LITG; right inferior temporal gyrus, RITG; left superior parietal lobule, LSPL; right superior parietal lobule, RSPL; left occipital lobe, LOC; right occipital lobe, ROC; left hippocampus, LHIP; right hippocampus, RHIP) were defined to encompass the main clusters of activation in the group activation map for each experimental condition contrasted against the fixation baseline. The activation time course for each ROI was extracted separately for each participant, based on the normalized and smoothed images, which were high-pass filtered and had the linear trend removed. The time courses across ROIs were correlated, and Fisher's *r* to *z* transformation was applied to the correlation coefficients prior to averaging and performing statistical comparison. In addition to the functional connectivity analysis described here, principal component analysis (PCA) was also used in SPSS (SPSS Inc. Chicago, IL) to examine the integration among regions. This method has been previously used to find connectivity of specialized areas of the visual cortex analogous to the established functional and anatomical distinctions (Ecker et al. 2007).

## Results

The main results of this study can be summarized as: (1) behavioral data showed that the participants were significantly faster and more accurate in locating the position of objects than in identifying them; (2) while the location detection task elicited greater activation in the dorsal visual stream, recognizing objects showed greater recruitment of the left ITG and the left IFG; (3) functional connectivity revealed stronger connection between ITG and occipital areas in object recognition task and between dorsal and ventral regions in location detection task; and (4) a PCA based on the correlation of the fMRI time course of activation between functional ROIs revealed three major components: frontoparietal, occipitotemporal, and subcortical.

### Behavioral data

Paired samples *t*-tests revealed a statistically significant difference in the mean reaction time for the location detection (M = 2158.93 msec, SD = 553.92 msec) and the object recognition (M = 2594.22 msec, SD = 420.77 msec) tasks, *t*(21) = 8.801, *P* < 0.001. A paired samples *t*-test was also used to examine performance accuracy in object and location tasks. This showed a statistically significant difference in accuracy during the location detection (M = 99.24%, SD = 1.6%) and object recognition (M = 93.56%, SD = 2.8%) tasks, *t*(21) =−4.55, *P* < 0.001.

### Brain activation

When object recognition and location detection tasks were contrasted with fixation baseline, a set of dorsal and ventral regions along with frontal and subcortical regions showed significant activation (*P* < 0.05, family-wise error corrected) (see Table 1 for a detailed list of peak locations and cluster size).

**Table 1:** Clusters of peak activation (MNI coordinates) in object recognition and location detection tasks contrasted with fixation baseline (family-wise error corrected threshold of *P* < 0.05).

Location versus fixation contrast						

Location of peak activation	*x*	*y*	*z*	Cluster	*t*-value	*P*-value
L middle occipital gyrus	−28	−96	12	9545	18.81	0.000
R thalamus	22	−28	6	321	15.34	0.000
L extra-nuclear	−22	−28	−4	198	13.43	0.000
L dorsolateral prefronatl cortex	−54	12	30	258	12.56	0.000
L middle frontal gyrus	−30	6	58	44	9.29	0.003
R cingulate gyrus	8	22	40	196	9.03	0.004
R fusiform gyrus	48	−64	−18	98	8.92	0.005
R precuneus	20	−62	46	130	8.8	0.006
R culmen/cerebellum	36	−40	−26	119	8.69	0.007

Object versus fixation contrast						
Location of peak activation	*x*	*y*	*z*	Cluster	*t*-value	*P*-value
L middle occipital gyrus	−30	−98	12	8771	25.32	0.000
L inferior frontal gyrus	−48	6	30	236	12.14	0.000
R thalamus	24	−28	−2	203	10.96	0.000
L extra-nuclear	−24	−30	−4	190	10.79	0.000
L superior parietal lobule	−30	−56	58	257	9.92	0.001
L thalamus	−16	−18	6	50	8.28	0.009
R cingulate gyrus	8	16	44	37	8.19	0.010
R subgyral	34	−66	−6	27	7.85	0.016
R culmen/cerebellum	36	−40	−26	9	7.71	0.020
R culmen/cerebellum	34	−50	−24	11	7.69	0.020
LIFG pars opercularis	−48	12	14	7	7.66	0.021
R subgyral	38	−54	−12	18	7.53	0.025

**Figure 2:**
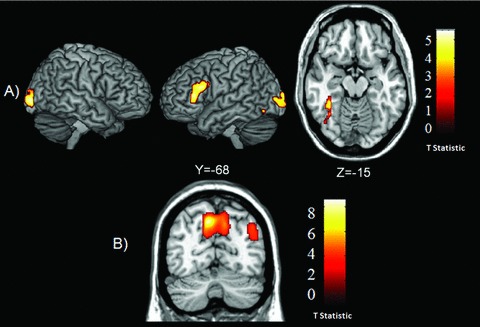
(A) Increased activation in bilateral occipital, left inferior frontal areas (surface rendering), and left inferior temporal lobe for object recognition relative to locating the position of objects. (B) Increased activation in bilateral precuneus and right angular gyrus while locating objects relative to recognizing them (*P* < 0.001). An extent threshold of 88 2-mm^3^ voxels was determined using 10,000 Monte Carlo simulations in AFNI to correct for false positives.

A direct comparison between object recognition and location detection tasks revealed differential recruitment of areas associated with visual and object processing. Participants showed significantly greater activation in bilateral precuneus (Left Precuneus: *x*=−10, *y*=−66, *z*= 44; BA (Brodmann Area) = 7; Right Precuneus: *x*= 10, *y*=−68, *z*= 42; BA = 7) (*P* < 0.001 with an extended threshold of 160 contiguous 2-mm^3^ voxels determined by Monte Carlo simulation) when they were detecting the location of objects (location > object) suggesting an increased visuospatial involvement in accomplishing this task. We also found greater activation in another parietal region, the right angular gyrus (*x*= 42, *y*=−74, *z*= 36; BA = 19), during location detection (see Fig. 2). The object recognition task (object > location), on the other hand, revealed significantly greater activation in the right middle occipital gyrus: *x*= 26, *y*=−94, *z*= 14, BA = 18; left middle occipital gyrus: *x*=−30, *y*=−98, *z*= 12, BA = 19; LITG: *x*=−38, *y*=−44, *z*=−14, and in the left inferior frontal gyrus (LIFG, *x*=−54, *y*= 32, *z*= 20, BA = 46). In other words, the object recognition task activated a wider network of occipitotemporal and frontal areas.

### Functional connectivity

**Figure 3:**
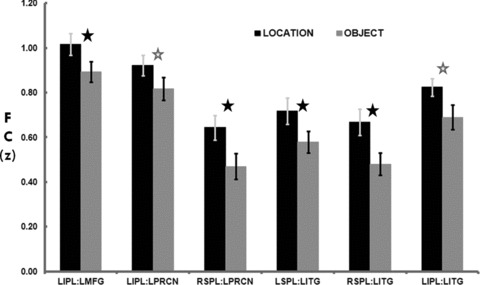
Functional connectivity differences between the two tasks. The first three bars indicate frontal–parietal connections, where as the rest indicate dorsal–ventral connections. Significant differences are indicated by dark stars.

The time course of activated voxels extracted from functional ROIs (mentioned earlier) was correlated to examine the functional connectivity across different brain areas. Several ROI pairs were found to have significantly different correlations when compared by condition (see Fig. 3). There was significantly greater connectivity between the frontal and parietal regions (LMFG and LIPL, *t*(21) = 2.65, *P*= 0.01; LPRCN and RSPL, *t*(21) = 2.00, *P*= 0.05; and LMFG and RSPL, *t*(21) = 2.12, *P*= 0.05) for the location detection task. There was also increased connectivity between the dorsal and ventral system ROIs during location detection task (LSPL and LITG, *t*(21) = 1.97, *P*= 0.05; RSPL and LITG, *t*(21) = 1.97, *P*= 0.05; and LIPL and LITG, *t*(21) = 1.86, *P*= 0.07). The differences in functional connectivity also approached significance in occipitotemporal connections in two ROI pairs for the object recognition task, LOC and RITG, *t*(21) = 1.94, *P*= 0.07, and LOC and LITG, *t*(21) = 1.86, *P*= 0.08. It should be noted that these effects are at a statistical threshold without multiple comparisons and none survived a multiple comparisons correction at a *P*-value of 0.0004. It is also possible that at this stringent correction, there is a good chance of type II error.

In order to examine the functional connectivity at the network level, a PCA of the *z*-transformed correlations of the time courses of the ROIs was conducted. This analysis revealed three components: *frontoparietal*, *subcortical*, *and occipitotemporal* networks (see Table 2). For experimental tasks (object recognition and location detection), the first three principal components accounted for 67% of the variance with the first factor being the largest, accounting for 41% of the variance. The second factor accounted for 14%, and the third for 12% of the variance in the model. The components revealed from this analysis indicate a possible division of labor with the ventral stream areas working together as a group (occipitotemporal component), the dorsal stream areas grouped with frontal areas (frontoparietal component) as another network, and the subcortical areas as yet another group.

**Table 2:** The three components extracted by the principal component analysis. Eigen values are included along with each component's associated variance accounted for by the model. ROIs listed by order of appearance are as follows: left inferior parietal lobule, right inferior parietal lobule, left superior parietal lobule, right superior parietal lobule, left middle frontal gyrus, medial prefrontal cortex, left precentral gyrus, supplementary motor area, left thalamus, right thalamus, right hippocampus, left occipital lobe, right occipital lobe, left inferior temporal gyrus, and right inferior temporal gyrus.

Component	ROI	Eigen value	% Variance	Cumulative
Frontoparietal	LIPL, RIPL, LSPL, RSPL, LMFG, MPFC, LPRCN, and SMA	1.85	40.86	40.86
Subcortical	LTHAL, RTHAL, RHIP	1.10	14.39	55.25
Occipitotemporal	LOC, ROC, LITG, RITG	1.00	11.96	67.21

## Discussion

The primary aim of this fMRI study was to examine the differential role of dorsal and ventral visual streams and their integrative functioning in tasks of object recognition and location detection. Our findings seem to support both specialization of the dorsal and ventral visual systems at one level, and the integration of these areas at another level. Based on previous findings ascribing specialized roles to dorsal and ventral stream areas in location detection and object recognition tasks, respectively, we predicted activation differences between the two tasks. We found significantly increased recruitment of three dorsal stream regions, right angular gyrus and bilateral precuneus, when participants detected the locations of objects. The role of precuneus in visuospatial processing (Cavanna and Trimble 2006), specifically in spatial attention as well as in shifting attention between object features and orientation of objects might be critical in performing this task (Le et al. 1998; Nagahama et al. 1999). Since the participants were asked to detect the location of an object relative to a cross in this task, they have to shift attention constantly as the locations keep changing from trial to trial. Such focusing and reorienting of visuospatial attention may also be reflected by greater activation found in the right angular gyrus (Rosenthal et al. 2009). The role of right angular gyrus in visuospatial attention has also been confirmed by studies using transcranial magnetic stimulation (Cattaneo et al. 2009). It is possible that an important aspect of locating the position of an object in space may be orienting and shifting attention. A previous study found anterior and posterior intraparietal sulcus to be most critical in distinguishing surface boundaries in three-dimensional (3D) space (Shikata et al. 2001). Although the present study did not use 3D images, there are similarities between finding surface boundaries and detecting an object's position around a cross.

Recognizing objects was relatively more difficult and slower for participants in this study as evidenced from the behavioral data. Overall, the behavioral results suggest that the participants were significantly faster and more accurate in locating the position of objects than in detecting and naming them. The location task might have been relatively easier for participants since there were only four locations (left, right, above, and below) to detect (although presented randomly) in contrast to recognizing unique objects every time. As expected, the object recognition condition showed more activation in the LITG. In addition, we found significantly increased activation in LIFG, bilateral thalami, and in occipital regions during this task. The increased IT recruitment has been found in previous studies of object recognition (Kanwisher et al. 1996; Gerlach et al.; Pietrini et al. 2004). Since the participants were asked to recognize an object and choose a name for it from four alternatives, they may engage in semantic characterizations of objects as reflected by the greater activation found in LIFG (Gabrieli et al. 1998; Hirsch et al.). In addition, word searches have also been found to activate the LIFG (Cornelissen et al. 2009). The results of this task revealed that recognizing objects may not be restricted to just the regions of the ventral visual stream, but may also include other cortical and subcortical regions. The thalamus has long been implicated in tasks of object naming in both schizophrenia (Heckers et al. 2000), and in typical individuals (Price et al. 1996). The LIFG activation seen in this task suggests the involvement of language, especially semantic characterization of objects. In addition, LIFG has also been specifically associated with tasks of covert object naming (Reed et al. 2004), selection of semantic information among competing alternatives (Thompson–Schill et al. 1998; Thompson–Schill et al. 2002; Kan and Thompson–Schill 2004), and in controlled retrieval of semantic knowledge (Wagner et al. 2001; Gold and Buckner 2002; Badre and Wagner 2004; Gold et al. 2005). Thus, our findings suggest that the object recognition task may recruit regions beyond the classic ventral stream areas.

Although the activation results, at least in part, might support specialized roles for the dorsal and ventral stream areas in these tasks, it is worth considering how these identified areas coordinate with other centers. For instance, the functional and causal interactions of dorsal and ventral visual stream areas were demonstrated to be important in learning tasks (Buchel et al. 1999). The precentral gyrus has been indicated in attention tasks in both schizophrenia and in attention deficit disorders (Dickstein et al. 2006; Dibbets et al. 2010; Sepede et al. 2010). The middle frontal gyrus has also been implicated in top-down attentional control for patients with Alzheimer's disease (Neufang et al. 2011). Increased connectivity between frontal (LMFG and LPRCN) and parietal (RSPL) regions during location detection may point to the demands in coordinating attention between the possible automatic identification of an object and then locating the position of that object. This is because the location detection in our study is sort of a dual task with simultaneously identifying the object while detecting the locations of them. In addition to the frontal–parietal connections, there was significantly increased connectivity between dorsal and ventral system regions during location detection task. This may suggest some level of integration of these two systems in tasks of location and object recognition.

This integration of frontal and parietal regions in location detection was also supported by evidence from the PCA analysis. The frontoparietal component (which accounted for maximum variance) seems to play a vital role in both these tasks. This may suggest that the locations and identities of the objects processed in the visual cortex may be elaborated in the parietal areas that further interact with the frontal areas. This evidence seems to be in line with the parietal–frontal integration theory (P-FIT), the premise of which involves the visual areas doing the work of perceiving the environment and then feeding that information forward to parietal areas that interact with frontal areas in making decisions about the processed information (Jung and Haier 2007). In our PCA analysis, the dorsal stream regions were grouped with frontal regions, suggesting a potential frontoparietal synchrony, and despite some evidence for specialization, the dorsal and ventral visual streams were not completely separated. In this way, the PCA results provide some preliminary evidence for the integration of dorsal, ventral, and other areas during object recognition and location detection.

The findings of this fMRI study provide further support for the role of dorsal and ventral visual streams in locating the positions of objects and in identifying them, respectively. Although lesion studies in monkeys have previously found evidence for this segregation, the present study also sheds light on to the integrative functioning of these streams with each other and with frontal and subcortical regions in accomplishing these tasks. Such integration may be a characteristic feature in human information processing as human perception is likely a conglomerate of external stimuli and self-derived expectations (Mesulam 2008). Thus, the rich experience driven knowledge base of humans may prompt the interpretation of a visuospatial or cognitive task to draw resources from multiple centers calling for integration among brain regions. Overall, this study suggests possible interactions between areas beyond the visual cortex that may play a role in visual processing and that there are spontaneous reactions to stimuli that begin on a general level and become increasingly more specific. Although the division of labor between dorsal and ventral visual streams may be limited to relatively posterior areas of the brain, such areas seem to communicate with frontal, as well as subcortical areas in accomplishing tasks of locating object positions and recognizing objects.
